# ATF6 activation promotes tumorigenesis and drug resistance in diffuse large B-cell lymphoma (DLBCL) by regulating the mTOR/S6K signaling pathway

**DOI:** 10.1007/s12672-025-02264-1

**Published:** 2025-04-09

**Authors:** Shuang Su, Lili Wu, Chen Huang, Cuiying He, Lianjing Wang, Weijing Li, Wei Liu, Lihong Liu

**Affiliations:** 1 Department of Hematology, The Fourth Hospital of Hebei Medical University, Shijiazhuang, 050011 China; 2 Hebei Provincial Key Laboratory of Tumor Microenvironment and Drug Resistance, The Fourth Hospital of Hebei Medical University, Shijiazhuang, 050011 China

**Keywords:** Diffuse large B-cell lymphoma, ATF6, CeapinA7, Oncogene, Therapeutic target

## Abstract

This study analyzes the expression and functional role of activating transcription factor 6 (ATF6) in diffuse large B-cell lymphoma (DLBCL) and its effects on disease progression. ATF6, a core component of the unfolded protein response (UPR) pathway, participates in many cellular activities and notably contributes to tumorigenesis. Through a combination of techniques, including immunohistochemistry (IHC) staining to assess ATF6 and pS6K levels, siRNA-mediated ATF6 knockdown, cytotoxicity assays, flow cytometry, quantitative real-time PCR (qRT-PCR), as well as Western blotting, this study clarified the functioning mechanisms of ATF6 in DLBCL and its potential clinical relevance. Further exploration of ATF6's involvement in the mTORC1 pathway was achieved through RNA sequencing (RNA-seq) and gene set enrichment analysis (GSEA). Our findings demonstrate that ATF6 expression is upregulated in DLBCL and linked to poor prognosis, particularly in people aged over 60 with Ann Arbor stage III-IV disease, B symptoms, non-GCB subtype, an international prognostic index (IPI) score greater than 2, and extranodal involvement. Notably, the ATF6 inhibitor ceapinA7 was shown to suppress ATF6 and mTORC1 activation, leading to less cell proliferation and the induction of apoptosis in DLBCL cells. Additionally, ceapinA7 increased the sensitivity of DLBCL cells to adriamycin. The foregoing results underscore the critical role of ATF6 in DLBCL and lay a theoretical and experimental foundation for future targeted therapies and drug development aimed at improving treatment outcomes for DLBCL.

**Supplementary Information:**

The online version contains supplementary material available at 10.1007/s12672-025-02264-1.

## Introduction

Diffuse large B-cell lymphoma (DLBCL), the most familiar non-Hodgkin lymphoma (NHL) subtype, features high aggressiveness and heterogeneity [1]. Although the first-line R-CHOP regimen has significantly ameliorated antitumor therapy efficacy, some patients remain insensitive to chemotherapy, experience rapid disease progression, and have poor prognosis [2]. Therefore, the identification of novel therapeutic approaches for patients with DLBCL is crucial.

Cancer cells maintain survival in the tumor microenvironment by activating several adaptive responses, notably unfolded protein response (UPR) [3]. UPR preserves endoplasmic reticulum (ER) homeostasis by promoting protein folding, translocation, and ER-related degradation, as well as mitigating protein synthesis, thereby boosting cancer cell growth and proliferation. UPR comprises three parallel signaling pathways: PERK- eIF2α, IRE1α- XBP1, as well as ATF6.

ATF6 is a pivotal signaling molecule in UPR. Under physiological conditions, ATF6 binds to BIP to form a complex in the ER. UPR activation begins with the BIP-bound dissociation of ATF6, which is subsequently translocated to the Golgi within the cell and cleaved by S1P and S2P proteases to yield a transcriptionally active form. This active ATF6 interacts with the ER stress response element (ERSE) in the promoter parts of target genes, thereby promoting their transcription. The resulting gene products are subsequently translated into proteins involved in protein folding processes [4]. After ER stress, ATF6 rapidly promotes BIP expression, which subsequently interacts with unfolded or misfolded proteins to mitigate ER stress. In normal conditions, BIP resides within the ER. In many cancers, its expression is elevated to detectable levels on the cell surface [5]. BIP expression is linked to cancer cell growth, histological classification, and outcomes related to tumor treatment and prognosis [6]. Additionally, UPR intersects with other adaptive responses crucial for cancer cell survival, such as autophagy [7, 8] and the DNA damage response (DDR) [9].

Several studies indicate that the expression of ATF6 is pivotal for tumor progression. In patients with colorectal cancer, increased ATF6 expression significantly correlates with decreased survival time and poor prognosis [10]. In dormant human squamous carcinoma cells (D-HEp3 cells), Rheb and mammalian target of rapamycin (mTOR) pathways were activated via ATF6. These pathways are being activated through the expression of ATF6. It helps cells adapt to both in vitro and in vivo chemotherapy microenvironment [11]. Furthermore, research has demonstrated that in human lymphoblastoid TK6 cells, ATF6 regulates autophagy and apoptosis via the mTOR pathway [12]. Knockdown of ATF6 decreased mTOR expression and enhanced autophagy and apoptosis. Although ATF6’s function in DNA damage repair has received relatively limited attention, studies have demonstrated that inhibiting ATF6, either pharmacologically or genetically, induces the degradation of BRCA1. This degradation exacerbates DNA damage and promotes cell death, thereby rendering colon cancer cells more sensitive to adriamycin [13]. Additionally, ATF6 can protect cardiac and neural tissues, which is also observed in diabetes models [14–19]. It acts as a crucial regulator of homeostasis, and its effects vary in specific cells and tissues. Dysregulated ATF6 signaling pathways possibly promote the pathogenesis of many illnesses, including cancer. However, the functional role of ATF6 in the pathogenesis of DLBCL, particularly its contribution to treatment resistance and crosstalk with the DNA damage response (DDR), is not yet fully elucidated. Therefore, this study endeavors to investigate the clinical relevance of ATF6 overexpression in DLBCL and its association with high-risk clinicopathological features, elucidate the mechanisms through which ATF6 promotes DLBCL cell survival and chemoresistance, and evaluate the therapeutic potential of the selective ATF6 inhibitor, ceapinA7, in inhibiting lymphomagenesis and overcoming adriamycin resistance.

This study presents the hypothesis that ATF6 may drive the progression of DLBCL by regulating the interplay between ER stress and genomic instability for the first time. This proposition not only broadens the theoretical understanding of the UPR in lymphoma biology but also lays the groundwork for the clinical translation of UPR-targeted therapies.

## Materials and methods

### In silico analysis

The microarray datasets GSE32918 and GSE83632 were retrieved from the Gene Expression Omnibus (GEO). The latter was utilized for differential expression analysis through the classical Bayesian approach using the Limma package before the ATF6 gene expression values were extracted.

### Cell lines and reagents

Human DLBCL cell lines LY3 and DHL6 were cultivated in RPMI 1640 (Gibco, USA) along with 10% fetal bovine serum (FBS, Cellmax, China). LY7 cells were supplemented with 10% FBS in IMDM (Gibco, USA) culture media. The aforementioned cells were kept at 37 °C with 5% CO_2_. CeapinA7 (sml2330) was obtained from Sigma-Aldrich. CQ (HY-17589) and adriamycin (HY-15142) were acquired from MCE.

### ATF6 silencing

ATF6 was knocked down by siRNA transfection in a human DLBCL cell line. Cells were inoculated and transfected with siRNA using CALNPTM RNAi in vitro (D-Nano Therapeutics, China) transfection reagent under the manufacturer’s guidelines. A generic and nonsensical RNA sequence was employed as the negative control siRNA. Details are presented in supplementary methods.

### Cytotoxicity assay

The cell viability was evaluated via Cell Counting Kit-8 assay. All DLBCL cell suspensions treated with drugs were injected in plates with 96 wells for 24–48 h. Details are provided in the Supplementary methods.

### RNA sequencing

RNA-easy™ Isolation Reagent(R701, Vazyme, China) was utilized to separate total cellular RNA. Total RNA (1 μg) was extracted from every sample, comprising three samples of LY3 cells treated with 20 μM CEAPINA7 and three samples treated with DMSO. The RNA sequencing (RNA-seq) transcriptome library was subsequently prepared via the Illumina® Stranded mRNA Prep kit (San Diego, CA). Paired-end sequencing of the RNA-seq library was performed on a NovaSeq 6000 sequencer, with sequencing services provided by Meiji Biotechnology (Shanghai, China).

### Immunohistochemical (IHC) staining

Paraffin blocks of the patient tissues were sectioned, deparaffinized, hydrated, and boiled in an autoclave for antigen repair. IHC staining was performed using primary antibodies specific for ATF6 (ab203119, Abcam) and pS6K (ab2571, Abcam). Next, secondary antibody incubation and DAB staining were performed. Details are shown in supplementary methods.

### Flow cytometry analysis

Apoptosis in the treated DLBCL cells was analyzed via flow cytometry. Details are shown in supplementary methods.

### Quantitative real-time PCR

Total RNA was isolated through RNA-easy™ Isolation Reagent (Vazyme, China). Reverse transcription was enabled by Reverse Transcription Reagent (Vazyme, China), followed by amplification via SYBR Green Premix (Vazyme, China) on a CFX Connect™ system (Bio-Rad, USA).

### Western blotting

Whole-cell protein lysates were extracted and investigated via western blotting as per established protocols [20]. Regarding protein expression assessment, the primary antibodies were rabbit polyclonal anti-ATF6 (ab203119, Abcam), anti-BiP/GRP78 (WL03157, Wanleibio), anti-S6K (ab32359, Abcam), anti-pS6K (ab2571, Abcam), anti-P62 (18420-1-AP, Proteintech), anti-LC3 (14600-1-AP, Proteintech), anti-γH2AX (HY-P80821, MCE), and mouse monoclonal anti-β-actin (HY-P80438, MCE). Goat anti-mouse IgG-HRP (RGAM001, Proteintech) and goat anti-rceapinA7it IgG-HRP (HY-P8001, MCE) were used as the secondary antibodies. Details are shown in supplementary methods.

### Statistical analysis

Every statistical analysis was completed through SPSS 20.0, while the figures were constructed through GraphPad Prism 8.0.2. Data obtained from three or more independent experiments are reported in mean ± standard deviation (SD). The survival curves were generated via Kaplan–Meier (KM) analysis, and group comparisons were conducted via the log-rank test. The correlation between the patient’s clinical characteristics and ATF6 expression was assessed by the continuity-corrected chi-square tests. The differences across groups were analyzed either via t-tests or one-way ANOVA. P < 0.05 suggested statistical significance.

## Results

### ATF6 expression is upregulated in DLBCL, which is linked to a poor prognosis

**Fig. 1:**
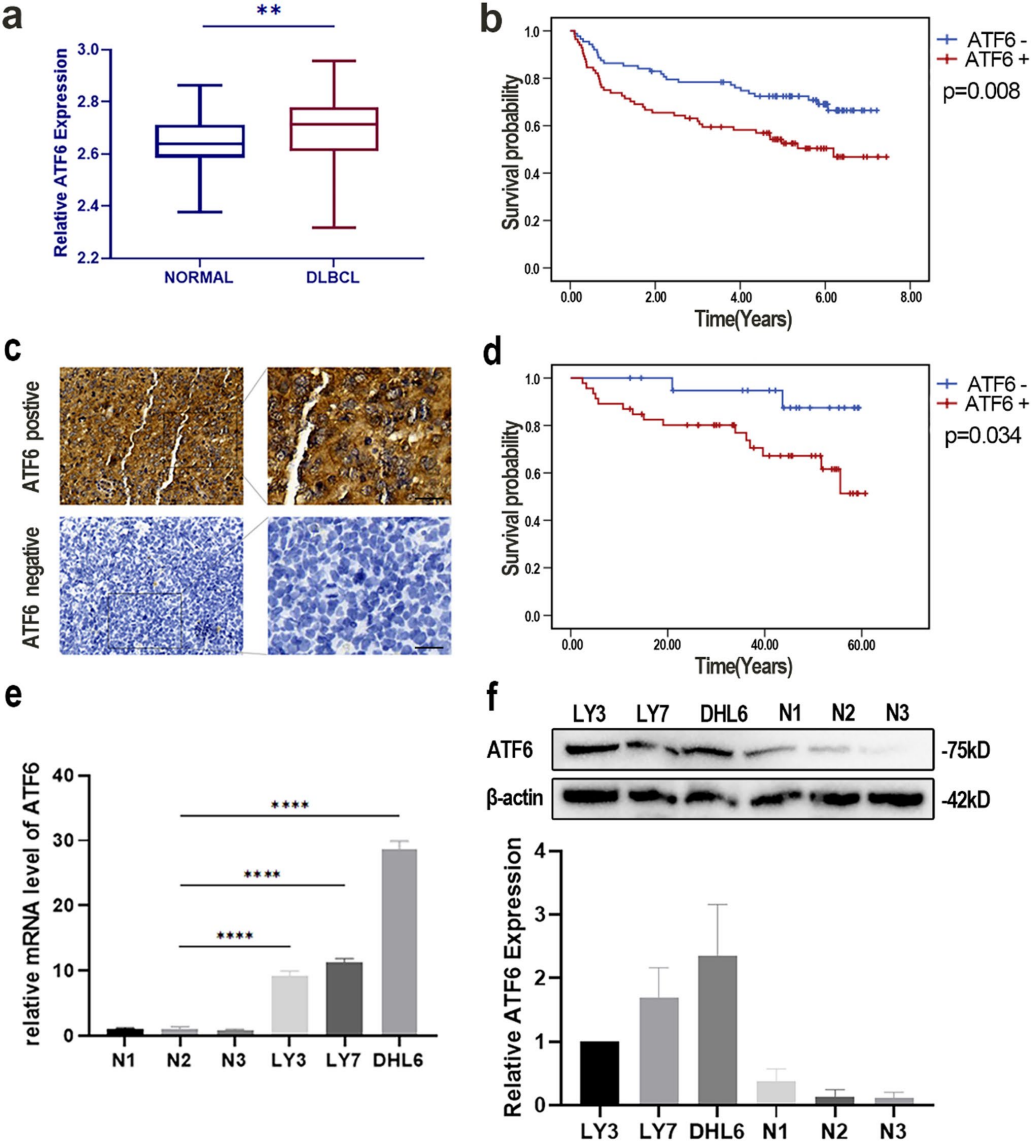
ATF6 expression is elevated in DLBCL, which is associated with poor prognosis. **a** ATF6 was up-regulated in DLBCL (n = 163) in contrast to normal within the GSE3283632 dataset. **b** K-M survival curve analysis revealed the link of high ATF6 expression in DLBCL to decreased OS in the GSE32918 dataset (n = 249, p = 0.008). **c** Representative IHC images of ATF6 staining of tumor tissue from patients with DLBCL (bar = 20 μm). **d** KM survival curve analysis demonstrated that increased ATF6 expression in DLBCL was linked to shortened OS (n = 67, p = 0.034) based on IHC data analysis. **e** Compared to control cells (N1, N2, N3), DLBCL cell lines (LY3, LY7, DHL6) exhibited significantly higher levels of ATF6 mRNA expression. **f** Western blotting assays confirmed elevated ATF6 protein expression in DLBCL cells. ****p < 0.0001

The significance of ATF6 in DLBCL was assessed by comparing its expression in DLBCL patients to normal controls, utilizing data from the GEO database. Bioinformatics analysis of the GSE83632 dataset, as depicted in Fig. 1a, revealed a marked elevation of ATF6 expression in the DLBCL cohort (n = 163) in comparison to normal controls. KM survival curve analysis of the GSE32918 dataset further demonstrated a significant link between elevated ATF6 expression and diminished overall survival (OS) (p = 0.008), as shown in Fig. 1b. IHC staining for ATF6 was performed on paraffin-embedded tissue samples from DLBCL patients diagnosed from 2019 to 2022 at the Department of Hematology, the Fourth Hospital of Hebei Medical University. 67 paraffin-embedded tissue samples from DLBCL patients were stained with IHC. The cohort included 30 males and 37 females, with a median age of 62 years (range: 19–82 years), and 38 patients (56.7%) were aged over 60 years. According to the Ann Arbor staging system, 13 patients were in stage I, 15 in stage II, 15 in stage III, and 24 in stage IV. Moreover, 26 patients presented with B symptoms. IPI scores were as follows: 12 patients scored 0–1, 20 patients had a score of 2, 17 patients scored 3, and 18 patients scored 4–5 (Table 1).

**Table 1:** Correlation of ATF6 expression with clinical characteristics of the DLBCL cohort

Characteristics	No. of patients	ATF6					*P*
		Positive(%)		Negative(%)			
Gender							
Male	30	22	73.33	8	26.67		
Female	37	24	64.86	13	35.14		0.457
Age(years)							
≤ 60	29	16	55.17	13	44.83		
> 60	38	30	78.95	8	21.05		**0.038**
Ann Arbor stage							
I–II	28	15	53.57	13	46.43		
III–IV	39	31	79.49	8	20.51		**0.024**
B symptoms							
Present	26	21	80.77	5	19.23		
Absent	41	25	60.98	16	39.02		0.089
Hans type							
GCB	19	11	57.89	8	42.11		
Non-GCB	48	35	72.92	13	27.08		0.232
ECOG score							
< 2	49	33	67.35	16	32.65		
≥ 2	18	13	72.22	5	27.78		0.703
Ki-67							
≥ 60%	54	38	70.37	16	29.63		
< 60%	13	8	61.54	5	38.46		0.538
LDH							
Normal	36	23	63.89	13	36.11		
Elevated	31	23	74.19	8	25.81		0.365
Extranodal involvement							
Present	41	32	78.05	9	21.95		
Absent	26	14	53.85	12	46.15		**0.037**
β2-MG							
Normal	45	31	68.89	14	31.11		
Elevated	22	15	68.18	7	31.82		0.953
IPI score							
< 2	18	9	50.00	9	50.00		
≥ 2	49	37	75.51	12	24.49		**0.046**
Double-expressor / double-hit							
Yes	34	23	67.65	11	32.35		
No	33	23	69.70	10	30.30		0.856
Lymphocyte							
< 1.1 × 10^9^/L	26	20	76.92	6	23.08		
Normal	41	26	63.41	15	36.59		0.245
pS6K							
Positive	37	35	94.59	2	5.41		
Negative	30	11	36.67	19	63.33		** < 0.0001**

IHC staining revealed that 68.66% (46/67) of DLBCL tissue samples exhibited high ATF6 expression (Fig. 1c). An analysis of clinicopathological characteristics indicated that ATF6 expression was positively linked to age (p = 0.038), Ann Arbor stage (p = 0.024), presence of extranodal involvement (p = 0.037), and the International Prognostic Index (IPI) score (p = 0.046) (Table 1). KM survival curve analysis confirmed that elevated ATF6 expression correlated with lowered OS in the DLBCL cohort (p = 0.034) (Fig. 1d). mRNA and protein levels of ATF6 were notably elevated in DLBCL cell lines compared with control cells from healthy people (Fig. 1e, f). All foregoing findings suggest that ATF6 overexpression is a key factor in DLBCL prognosis.

### The ATF6 inhibitor ceapinA7 suppresses DLBCL proliferation and induces apoptosis

**Fig. 2:**
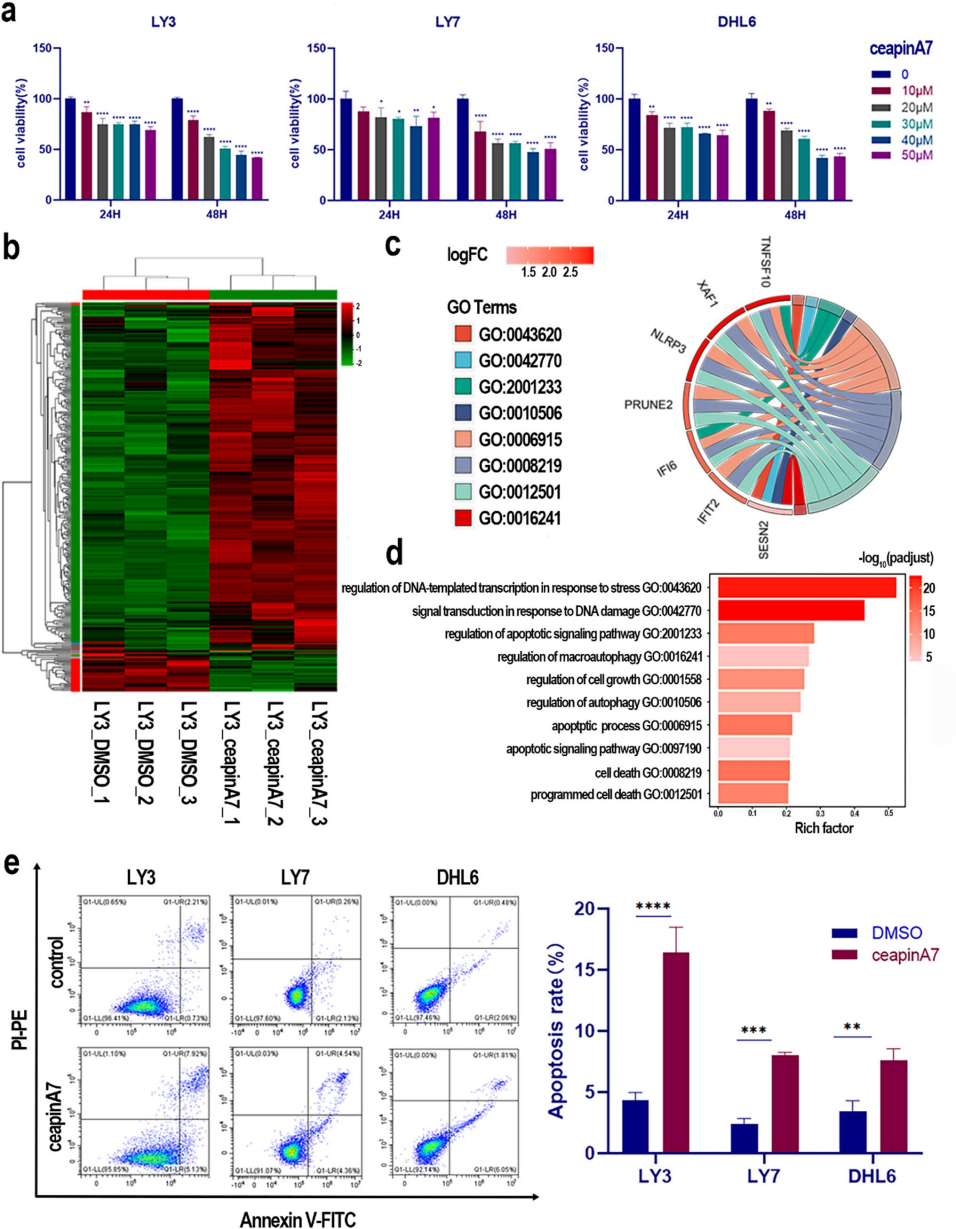
The ATF6 inhibitor ceapinA7 suppresses DLBCL proliferation and induces apoptosis. **a** CeapinA7 decreased the viability of DLBCL cell lines. **b** Heatmap showing the expression characteristics of relevant genes following ceapinA7 application in RNA-Seq analysis. **c** Functional enrichment of differentially expressed genes in upregulated groups in LY3 following ceapinA7 treatment in RNA-seq. **d** Description of the biological processes involved in gene enrichment. **e** CeapinA7 triggered apoptosis in DLBCL cell lines, as assessed through flow cytometry via Annexin V and PI staining

To further elucidate the effects of ATF6 on DLBCL, ATF6 inhibitors, a class of pyrazolamides, were leveraged to block ATF6α signaling during ER stress without affecting IRE1 and PERK activation [21]. According to Benedetti et al., ceapinA7 inhibits Burkitt's lymphoma cell activity and promotes apoptosis [22]. DLBCL cell lines (LY3, LY7, and DHL6) were treated with varying concentrations of ceapinA7 for durations of 24 to 48 h. The viability of DLBCL cells was also reduced (Fig. 2a). DLBCL cell activity decreased with increasing ceapinA7 concentration and exposure time. The role of ATF6 in DLBCL was explored in greater detail using RNA-seq analysis of LY3 cells treated with ceapinA7. Differential expression analysis of genes and transcripts indicated that ATF6 regulates DNA damage and cell death, as supported by annotations from GO analysis (Fig. 2b–d). The biological processes associated with ATF6 expression were subsequently confirmed in DLBCL cell lines. Annexin V-FITC/PI-PE apoptosis assay demonstrated that after 24 h of incubation with 20 μM ceapinA7, apoptosis levels in DLBCL cell lines LY3 (control: 4.33 ± 0.64% vs. ceapinA7 group: 16.42 ± 2.08%, P = 0.0007), LY7 (control: 2.37 ± 0.46% vs. ceapinA7 group: 8.00 ± 0.27%, P = 0.0001), and DHL6 (control: 3.47 ± 0.83% vs. ceapinA7 group: 7.60 ± 0.96%, P = 0.0049) were elevated (Fig. 2e), with the most significant effect observed in LY3.

### CeapinA7 promotes cellular autophagy by inhibiting mTORC1 activation

**Fig. 3:**
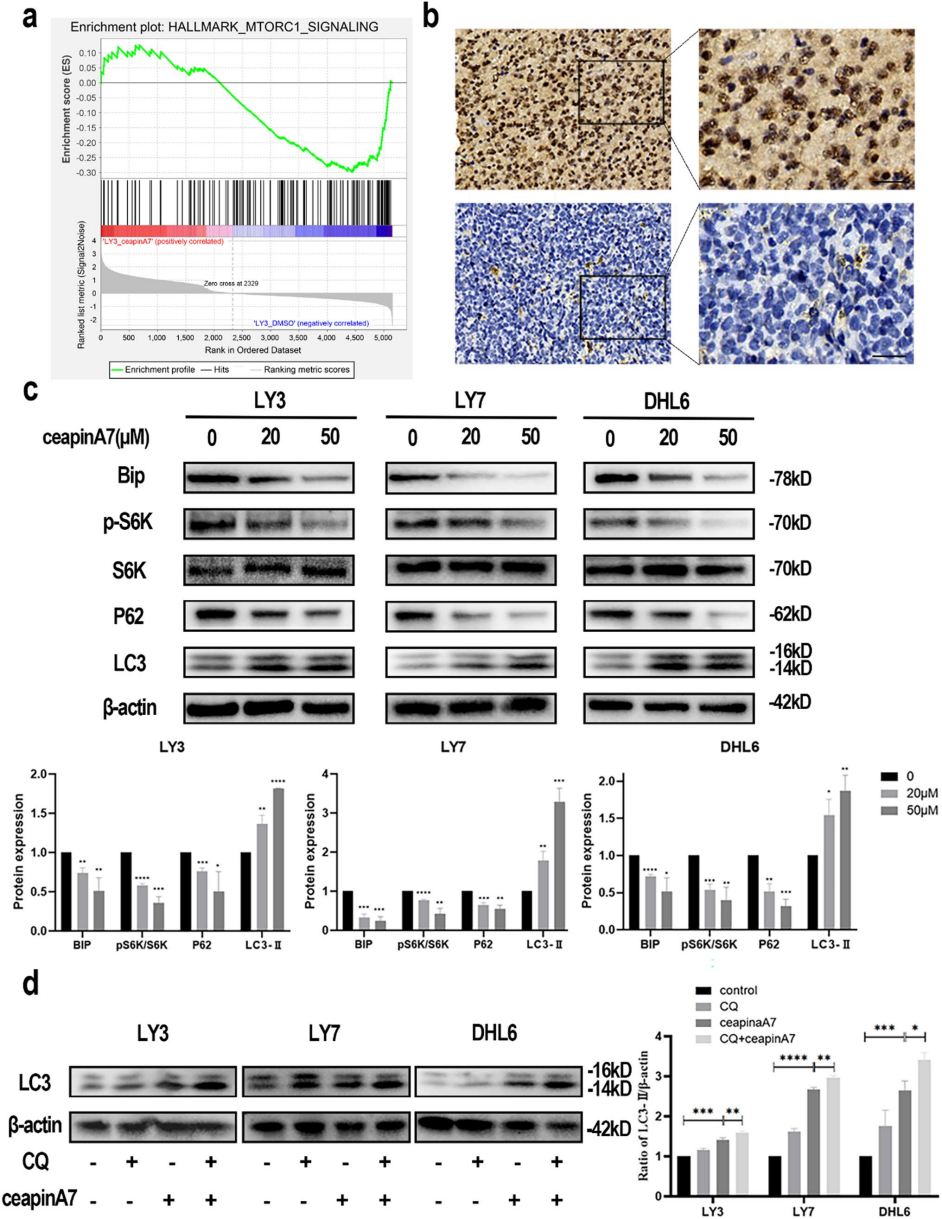
CeapinA7 promotes cellular autophagy by inhibiting mTORC1 activation. **a** GSEA based on RNA-seq proved significant enrichment of ATF6 in mTORC1. **b** Representative IHC images of pS6K staining of tumor tissue from patients with DLBCL (bar = 20 μm). **c** CeapinA7 induced S6K dephosphorylation and cellular autophagy protein expression in DLBCL cell lines. Cells were subject to the specified concentrations of ceapinA7, and the expression levels of Bip, phosphorylated S6K, P62, and LC3 proteins were evaluated through Western blot analysis, with β-actin as a loading control. Quantification was based on three independent experiments, with band intensities normalized to the control via ImageJ. Data are displayed in mean ± SD., n = 3. **d** WB detection of changes in LC3-II/β-actin expression after combined application of ceapinA7 and CQ. Results are shown as mean ± SD. p-values: * < 0.05; ** < 0.01; *** < 0.001; **** < 0.0001

The potential regulatory mechanisms of CeapinA7 in DLBCL cell lines were subsequently analyzed using GSEA based on RNA-seq data. Functional annotation highlighted the involvement of the mTORC1 signaling pathway (Fig. 3a). mTOR, a serine/threonine kinase, is a key modulator of cell growth and metabolism [23]. It forms two distinctive complexes in conjunction with several proteins: mTOR complex 1 (mTORC1) and mTOR complex 2 (mTORC2) [24]. The former is a signaling pathway responsible for various downstream components including ribosomal protein S6 kinase β-1 (p706K) as well as 4E-binding protein 1 (4E-BP1). mTORC1 activation phosphorylates its downstream substrates, thereby facilitating anabolic processes that drive the synthesis of ribosomes and essential biomolecules, including proteins, nucleotides, fatty acids, and lipids. Conversely, it suppresses catabolic pathways, such as autophagy [25, 26].

IHC staining of paraffin sections from 67 patients with DLBCL showed a high expression rate of pS6K at 55.22% (37/67) (Fig. 3b). In the pS6K high-expression group, the expression rate of ATF6 was 94.59% (35/37), whereas, in the low pS6K expression group, it was only 36.67% (11/30), which demonstrated a statistically significant variation across groups (P < 0.001). After ceapinA7 was applied to DLBCL cell lines, protein immunoblotting detected pS6K expression, which indicated that ceapinA7 inhibited S6K phosphorylation (Fig. 3c). The effect of ceapinA7 on ATF6 inhibition was assessed by evaluating the reduction in Bip, which is significantly influenced by ATF6 activity [27]. The levels of proteins associated with autophagy in CeapinA7-treated DLBCL cell lines were assessed via western blot analysis. The findings demonstrated that the formation of double-membrane autophagosomes necessitates an upregulation of LC3-II, the lipidated form of microtubule-correlated protein 1 light chain 3 (LC3), accompanied by a decrease in P62 protein levels (Fig. 3c). These results validate that CeapinA7 induces autophagy in DLBCL cell lines. Notably, chloroquine (CQ), an autophagy inhibitor, disrupts autophagic lysosome formation. The degree of LC3-II accumulation after CQ treatment indirectly reflects the changes in autophagic flux. To explore the occurrence of cellular autophagy, cells were co-treated with CQ (20 mM) and ceapinA7 (20 μM) for 24 h. LC3-II levels in the co-treated cohort were higher than those in the ceapinA7-treated cohort (Fig. 3d). Therefore, ceapinA7 treatment promotes autophagy in DLBCL cell lines.

### Silencing of ATF6 is consistent with the ceapinA7-induced increase in apoptosis and activation of autophagy in DLBCL cell lines

**Fig. 4:**
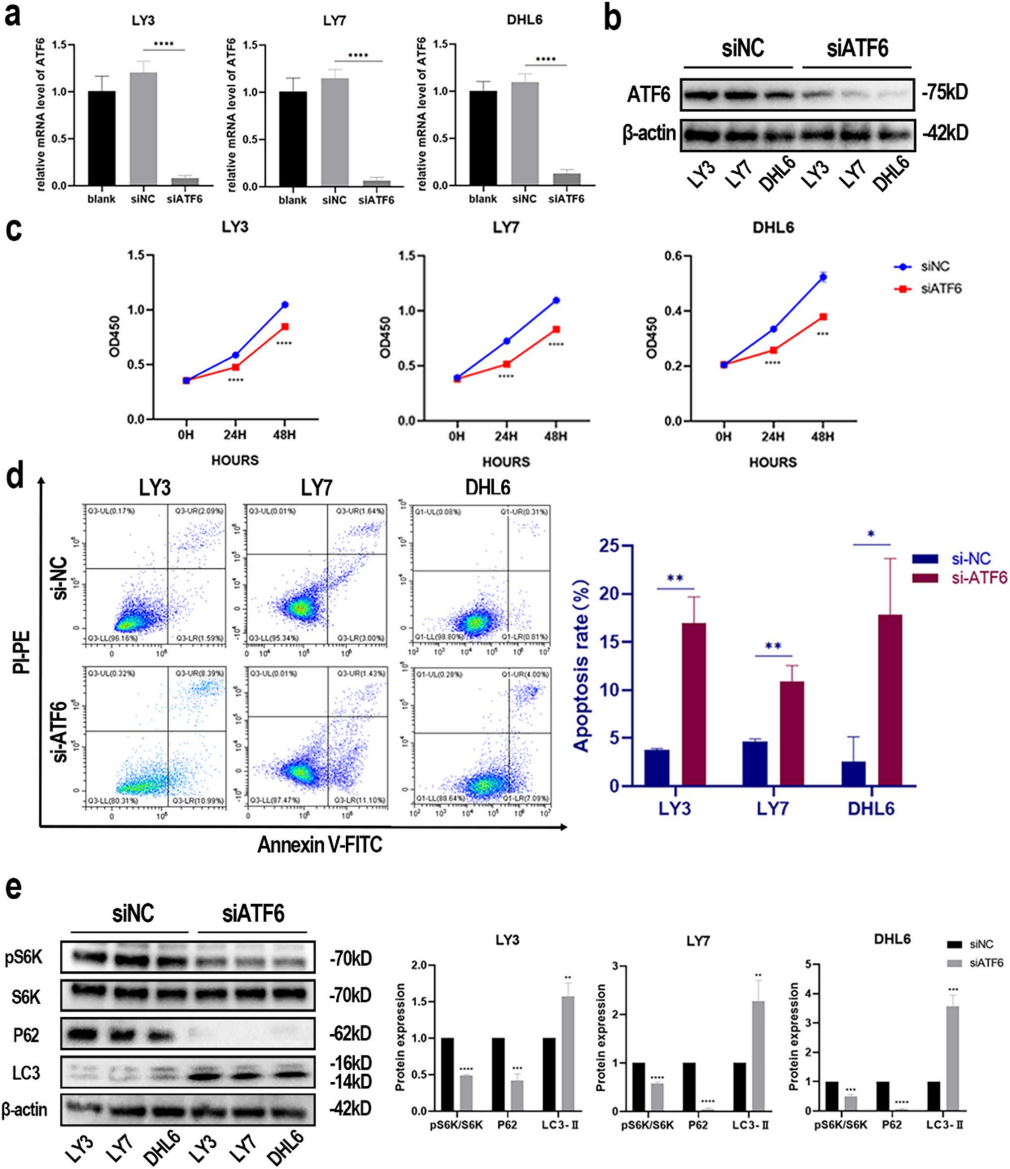
Silencing of ATF6 is consistent with the ceapinA7-induced increase in apoptosis and activation of autophagy in DLBCL cell lines. **a**, **b** Relative expression levels of ATF6 in DLBCL cells after transfection with siATF6 were assessed using qPCR (mean ± SD, n = 3) and western blotting in comparison to siNC. **c** ATF6 knockdown significantly decreased cell proliferation. **d** ATF6 knockdown triggered apoptosis in DLBCL cell lines assessed via flow cytometry using Annexin V and PI staining. **e** Western blotting assay for S6K phosphorylation, P62, and LC3-II expression changes after ATF6 knockdown. Data are displayed as mean ± SD. p-values: * < 0.05; ** < 0.01; *** < 0.001; **** < 0.0001

The ATF6 expression in DLBCL cell lines (LY3, LY7, and DHL6) was silenced using siRNA, and the knockdown effect was verified by western blotting and qRT-PCR (Fig. 4a, b). In comparison to siNC, siATF6 transfected cells exhibited growth inhibition (Fig. 4c). Annexin V/PI apoptosis assay results demonstrated that siATF6 induced apoptosis in DLBCL cell lines LY3 (3.78 ± 0.15% in control vs. 17.00 ± 2.67% in siATF6, P = 0.001), LY7 (4.63 ± 0.30% in control vs. 10.87 ± 1.69% in siATF6, P = 0.003), and DHL6 (2.58 ± 2.57% in control vs. 17.84 ± 5.83% in siATF6, P = 0.014) (Fig. 4d). Changes in S6K phosphorylation and autophagy-related indicators were verified in ATF6 knockdown DLBCL cell lines. S6K phosphorylation was lowered in the siATF6 group, where LC3-II was elevated and P62 was reduced (Fig. 4e). These results further prove that ATF6 inhibition promotes apoptosis and facilitates cellular autophagy by inhibiting the activation of mTORC1.

### CeapinA7 enhances chemosensitivity in DLBCL

**Fig. 5:**
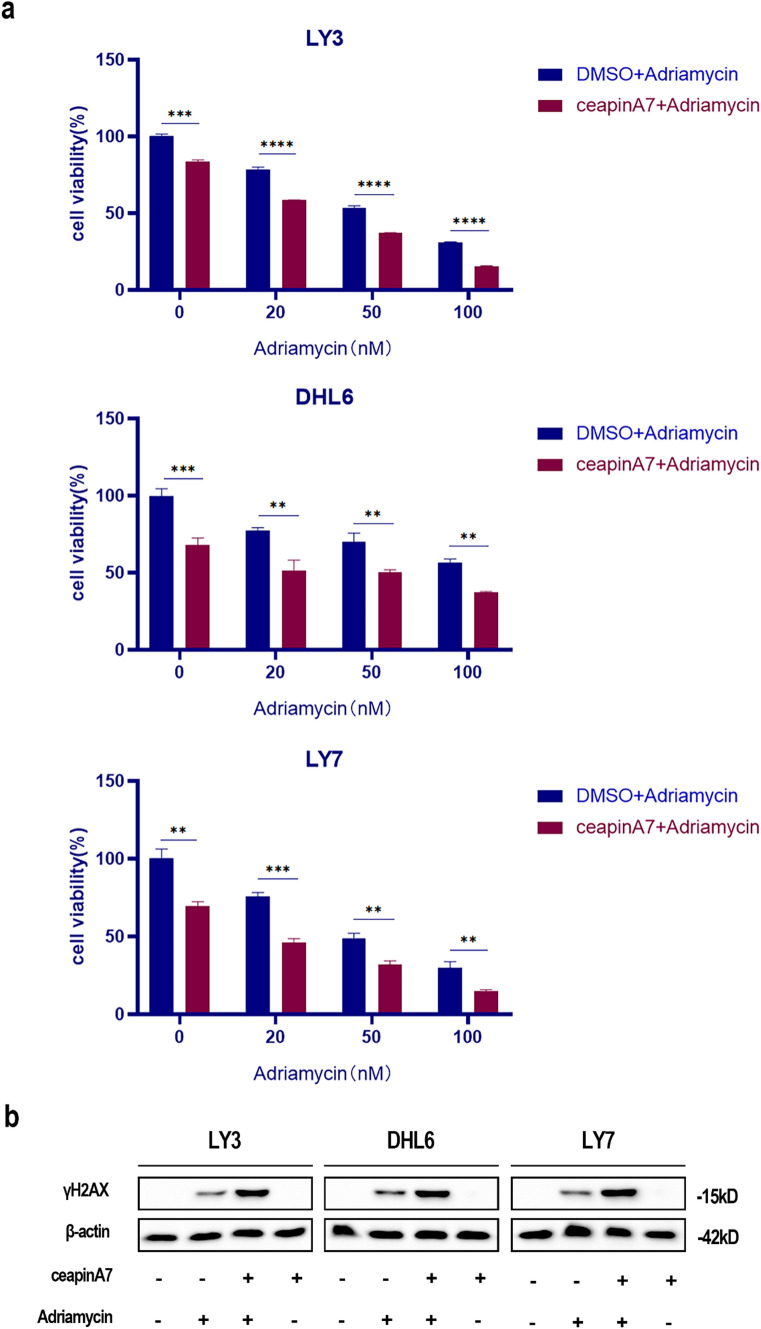
CeapinA7 Enhances Chemosensitivity in DLBCL. **a** DLBCL cells were co-treated with varying concentrations of adriamycin, with or without 20 μM ceapinA7, and subsequently subjected to a CCK8 assay after 48 h. Cell proliferation in DLBCL cell lines was significantly suppressed in the ceapinA7-treated group. Results are shown in mean ± SD, n = 3. **p < 0.01; ***p < 0.001; ****p < 0.0001. **b** Western blotting analysis was undertaken to assess the DNA damage marker γH2AX. Elevated γH2AX expression was observed after co-treatment of DLBCL cells with adriamycin and ceapinA7

Adriamycin, a DNA-damaging agent, represents a classic treatment method for DLBCL. This study assessed the potential of ceapinA7 to augment the cytotoxic efficacy of adriamycin. Results showed that DLBCL cell activity progressively decreased with increasing concentrations of adriamycin. Additionally, the cytotoxic effect was further improved while the cells were co-treated with ceapinA7 and adriamycin (Fig. 5a). Histone H2AX is a variant of H2A that replaces conventional H2A in nucleosomes. When DNA is damaged, the Ser139 site of histone H2AX is rapidly phosphorylated to form γH2AX, a sensitive marker of DNA damage. Subsequent analysis of γH2AX expression in cells subjected to the combinational treatment was completed utilizing the Western blot technique. As illustrated in Fig. 5b, the amount of γH2AX was greater in cells treated with both ceapinA7 and adriamycin than in the ones treated with adriamycin only. It can thus be inferred from these findings that ceapinA7 can indeed sensitize DLBCL cells to the cytotoxic effects of adriamycin.

## Discussion

Recent research on UPR has gained popularity, which reflects its crucial role in tumor growth and drug resistance. Specific signaling molecules within this pathway are regarded as promising targets for cancer therapy [4]. ATF6 has become the focal point of extensive research regarding its role in cancer progression. Increased expression of ATF6 has been observed in many tumor tissues in contrast to that in normal ones. Clinical sample analysis has demonstrated an upregulation of ATF6 in colorectal cancer [10], oral squamous cell carcinoma [28, 29], and gastric cancer [30], and its overexpression is detrimental to patient survival. Our analysis of publicly accessible cancer microarray databases and IHC staining of DLBCL paraffin sections from our research center confirmed that ATF6 expression is elevated in DLBCL and that this increase correlates with a reduced OS rate. Elevated ATF6 expression levels were significantly linked to advanced age, higher Ann Arbor stage, increased IPI scores, and extranodal involvement in DLBCL patients, all of which suggest a poorer disease prognosis. Similarly, there was a marked elevation in ATF6 expression in DLBCL cell lines in comparison to PBMCs from healthy donors. In conclusion, ATF6 may be a prognostic marker for DLBCL.

Regarding ATF6’s specific role in modulating tumor cell growth, the knockdown of ATF6 impedes cancer cell proliferation and migration while promoting autophagy and apoptosis in oral squamous cell carcinoma [28]. Pharmacological and genetic inhibition of ATF6 demonstrated that both ceapinA7 and siATF6 effectively inhibited the growth of DLBCL cell lines. This indicated that the inhibition of ATF6 could potentially function as a tumor suppressor, and RNA-seq analyses further indicated that regulating ATF6 may alter cancer cell responses to DNA damage, apoptosis, and autophagy. Our data suggest that ATF6 inhibition exerts a therapeutic effect on DLBCL cells by regulating DNA damage signaling pathways and activating apoptosis and autophagy. ATF6 expression possibly regulates DNA damage and thus influences tumor resistance to DNA-damaging agents [13]. ATF6 inhibition in high-grade plasmacytoid ovarian cancer further emphasizes its ability to enhance tumor sensitivity to chemotherapy [31]. CeapinA7 was noted to sensitize DLBCL cells to the cytotoxic effects of adriamycin, thereby increasing cellular DNA damage. These findings imply that ATF6 inhibition may boost the anticancer effects of DNA-damaging agents, with adriamycin being a common treatment for DLBCL. However, the precise regulatory mechanisms of ATF6 suppression in DNA damage response in DLBCL cells warrant further investigation, which will be the focus of future studies.

ATF6 ameliorates tumor survival by activating the mTOR pathway, facilitating adaptation to chemotherapy, and responding to in vivo microenvironment [11]. In human lymphoblastoid cells (TK6 cells), ATF6 knockdown gives rise to decreased mTOR expression and enhanced autophagy and apoptosis [12]. Gremlin-1 facilitates the metastasis of colorectal cancer cells by stimulating ATF6 and the PI3K/AKT/mTOR pathway [32]. Our RNA-seq-based GSEA analysis showed that ATF6 was functionally enriched in the mTORC1 signaling pathway, which aligns with past findings. CeapinA7 triggered apoptosis and autophagy in DLBCL cells. Western blot analysis confirmed that ceapinA7 significantly inhibited S6K phosphorylation, a downstream target of mTORC1, thus promoting autophagy. When CQ was added to inhibit late autophagy in DLBCL cell lines treated with ceapinA7, the effect of ceapinA7 on autophagy in DLBCL cells was further confirmed. The conclusion was further substantiated through ATF6 knockdown. The activation of S6K signaling induced by ATF6 may be pivotal for the development of DLBCL. More research is imperative to elucidate the underlying biological mechanisms and signaling interactions linked to ATF6 dysregulation. The effects of ceapinA7 on a DLBCL mouse model will be discussed in future studies.

Our study proves the pivotal role of ATF6 in the pathogenesis of DLBCL. ATF6 is significantly upregulated in DLBCL and strongly correlates with adverse patient prognosis. Mechanistically, ATF6 promotes cell proliferation and survival by activating the mTORC1/pS6K signaling pathway. Notably, the ATF6 inhibitor ceapinA7 exhibits potent antitumor effects by suppressing ATF6 activity. In addition, ceapinA7 enhances the sensitivity of DLBCL cells to adriamycin, which indicates its potential as an adjunctive therapy to overcome drug resistance.

The foregoing findings highlight the importance of exploring UPR-related pathways in cancer biology. Our study lays a robust experimental foundation for future investigations focused on developing ATF6 inhibitors or combination therapies, positioning ATF6 as a promising therapeutic target for DLBCL intervention.

## Supplementary Information

Supplementary Material 1.

## Data Availability

A reasonable request can be made to the corresponding author if you would like to access utilized and/or analyzed datasets.

## References

[1] Swerdlow SH, Campo E, Pileri SA, Harris NL, Stein H, Siebert R, Advani R, Ghielmini M, Salles GA, Zelenetz AD, Jaffe ES. The 2016 revision of the World Health Organization classification of lymphoid neoplasms. Blood. 2016;127:2375–90. 10.1182/blood-2016-01-643569. 26980727 10.1182/blood-2016-01-643569 PMC4874220

[2] Zhou N, Choi J, Grothusen G, Kim B-J, Ren D, Cao Z, Liu Y, Li Q, Inamdar A, Beer T, Tang H-Y, Perkey E, Maillard I, Bonasio R, Shi J, Ruella M, Wan L, Busino L. DLBCL-associated NOTCH2 mutations escape ubiquitin-dependent degradation and promote chemoresistance. Blood. 2023;142:973–88. 10.1182/blood.2022018752. 37235754 10.1182/blood.2022018752 PMC10656726

[3] Madden E, Logue SE, Healy SJ, Manie S, Samali A. The role of the unfolded protein response in cancer progression: from oncogenesis to chemoresistance. Biol Cell. 2018;111:1–17. 10.1111/boc.201800050. 30302777 10.1111/boc.201800050

[4] Wang M, Kaufman RJ. The impact of the endoplasmic reticulum protein-folding environment on cancer development. Nat Rev Cancer. 2014;14:581–97. 10.1038/nrc3800. 25145482 10.1038/nrc3800

[5] Arap MA, Lahdenranta J, Mintz PJ, Hajitou A, Sarkis ÁS, Arap W, Pasqualini R. Cell surface expression of the stress response chaperone GRP78 enables tumor targeting by circulating ligands. Cancer Cell. 2004;6:275–84. 10.1016/j.ccr.2004.08.018. 15380518 10.1016/j.ccr.2004.08.018

[6] Lee AS. GRP78 induction in cancer: therapeutic and prognostic implications. Cancer Res. 2007;67:3496–9. 10.1158/0008-5472.Can-07-0325. 17440054 10.1158/0008-5472.CAN-07-0325

[7] Lin Y, Jiang M, Chen W, Zhao T, Wei Y. Cancer and ER stress: mutual crosstalk between autophagy, oxidative stress and inflammatory response. Biomed Pharmacother. 2019. 10.1016/j.biopha.2019.109249. 31351428 10.1016/j.biopha.2019.109249

[8] Romeo MA, Gilardini Montani MS, Gaeta A, D’Orazi G, Faggioni A, Cirone M. HHV-6A infection dysregulates autophagy/UPR interplay increasing beta amyloid production and tau phosphorylation in astrocytoma cells as well as in primary neurons, possible molecular mechanisms linking viral infection to Alzheimer’s disease. Biochim Biophys Acta Mol Basis Dis. 2020. 10.1016/j.bbadis.2019.165647. 31866416 10.1016/j.bbadis.2019.165647

[9] González-Quiroz M, Blondel A, Sagredo A, Hetz C, Chevet E, Pedeux R. When endoplasmic reticulum proteostasis meets the DNA damage response. Trends Cell Biol. 2020;30:881–91. 10.1016/j.tcb.2020.09.002. 33036871 10.1016/j.tcb.2020.09.002

[10] Coleman OI, Lobner EM, Bierwirth S, Sorbie A, Waldschmitt N, Rath E, Berger E, Lagkouvardos I, Clavel T, McCoy KD, Weber A, Heikenwalder M, Janssen K-P, Haller D. Activated ATF6 induces intestinal dysbiosis and innate immune response to promote colorectal tumorigenesis. Gastroenterology. 2018;155:1539-1552.e1512. 10.1053/j.gastro.2018.07.028. 30063920 10.1053/j.gastro.2018.07.028

[11] Schewe DM, Aguirre-Ghiso JA. ATF6α-Rheb-mTOR signaling promotes survival of dormant tumor cells in vivo. Proc Natl Acad Sci U S A. 2008;105:10519–24. 10.1073/pnas.0800939105. 18650380 10.1073/pnas.0800939105 PMC2492459

[12] Li B, Yang H, Wu H, Wang H, Ye Z, Wu Y, Chen Y, Tang H. Hydroquinone-induced endoplasmic reticulum stress affects TK6 cell autophagy and apoptosis via ATF6-mTOR. Environ Toxicol. 2023;38:1874–90. 10.1002/tox.23814. 37148176 10.1002/tox.23814

[13] Benedetti R, Romeo MA, Arena A, Gilardini Montani MS, Di Renzo L, D’Orazi G, Cirone M. ATF6 prevents DNA damage and cell death in colon cancer cells undergoing ER stress. Cell Death Discov. 2022. 10.1038/s41420-022-01085-3. 35752616 10.1038/s41420-022-01085-3 PMC9233702

[14] Glembotski CC. Roles for ATF6 and the sarco/endoplasmic reticulum protein quality control system in the heart. J Mol Cell Cardiol. 2014;71:11–5. 10.1016/j.yjmcc.2013.09.018. 24140798 10.1016/j.yjmcc.2013.09.018 PMC4157898

[15] Yu Z, Sheng H, Liu S, Zhao S, Glembotski CC, Warner DS, Paschen W, Yang W. Activation of the ATF6 branch of the unfolded protein response in neurons improves stroke outcome. J Cereb Blood Flow Metab. 2016;37:1069–79. 10.1177/0271678x16650218. 27217380 10.1177/0271678X16650218 PMC5363481

[16] Naranjo JR, Zhang H, Villar D, González P, Dopazo XM, Morón-Oset J, Higueras E, Oliveros JC, Arrabal MD, Prieto A, Cercós P, González T, De la Cruz A, Casado-Vela J, Rábano A, Valenzuela C, Gutierrez-Rodriguez M, Li J-Y, Mellström B. Activating transcription factor 6 derepression mediates neuroprotection in Huntington disease. J Clin Invest. 2016;126:627–38. 10.1172/jci82670. 26752648 10.1172/JCI82670 PMC4731176

[17] Kezuka D, Tkarada-Iemata M, Hattori T, Mori K, Takahashi R, Kitao Y, Hori O. Deletion of Atf6α enhances kainate-induced neuronal death in mice. Neurochem Int. 2016;92:67–74. 10.1016/j.neuint.2015.12.009. 26724566 10.1016/j.neuint.2015.12.009

[18] Plate L, Cooley CB, Chen JJ, Paxman RJ, Gallagher CM, Madoux F, Genereux JC, Dobbs W, Garza D, Spicer TP, Scampavia L, Brown SJ, Rosen H, Powers ET, Walter P, Hodder P, Wiseman RL, Kelly JW. Small molecule proteostasis regulators that reprogram the ER to reduce extracellular protein aggregation. Elife. 2016. 10.7554/eLife.15550. 27435961 10.7554/eLife.15550 PMC4954754

[19] Usui M, Yamaguchi S, Tanji Y, Tominaga R, Ishigaki Y, Fukumoto M, Katagiri H, Mori K, Oka Y, Ishihara H. Atf6α-null mice are glucose intolerant due to pancreatic β-cell failure on a high-fat diet but partially resistant to diet-induced insulin resistance. Metabolism. 2012;61:1118–28. 10.1016/j.metabol.2012.01.004. 22386934 10.1016/j.metabol.2012.01.004

[20] Li W, Wu L, Huang C, Ma H, Wang L, Liu W, Liu L. Activation of Notch-1 signaling pathway in macrophages to secrete PD-L1 and regulate cytotoxicity of CAR-T cells in diffuse large B-cell lymphoma. Aging. 2024;16:1845–59. 10.18632/aging.205463. 38261741 10.18632/aging.205463 PMC10866421

[21] Gallagher CM, Garri C, Cain EL, Ang KK-H, Wilson CG, Chen S, Hearn BR, Jaishankar P, Aranda-Diaz A, Arkin MR, Renslo AR, Walter P. Ceapins are a new class of unfolded protein response inhibitors, selectively targeting the ATF6α branch. Elife. 2016. 10.7554/eLife.11878. 27435960 10.7554/eLife.11878 PMC4954757

[22] Benedetti R, Arena A, Romeo MA, Gilardini Montani MS, Gonnella R, Santarelli R, Trivedi P, Cirone M. Concomitant inhibition of IRE1α/XBP1 axis of UPR and PARP: a promising therapeutic approach against c-Myc and Gammaherpesvirus-driven B-cell lymphomas. Int J Mol Sci. 2022. 10.3390/ijms23169113. 36012375 10.3390/ijms23169113 PMC9409055

[23] Zhang Y, Chou SD, Murshid A, Prince TL, Schreiner S, Stevenson MA, et al. The role of heat shock factors in stress-induced transcription. Methods Mol Biol. 2011;787:21–32. 10.1007/978-1-61779-295-3_2. 21898224 10.1007/978-1-61779-295-3_2 PMC4088327

[24] Bhaskar PT, Hay N. The two TORCs and Akt. Dev Cell. 2007;12:487–502. 10.1016/j.devcel.2007.03.020. 17419990 10.1016/j.devcel.2007.03.020

[25] Kim YC, Guan K-L. mTOR: a pharmacologic target for autophagy regulation. J Clin Invest. 2015;125:25–32. 10.1172/jci73939. 25654547 10.1172/JCI73939 PMC4382265

[26] Castedo M, Ferri KF, Kroemer G. Mammalian target of rapamycin (mTOR): pro- and anti-apoptotic. Cell Death Differ. 2002;9:99–100. 10.1038/sj.cdd.4400978. 11840159 10.1038/sj.cdd.4400978

[27] Hillary RF, FitzGerald U. A lifetime of stress: ATF6 in development and homeostasis. J Biomed Sci. 2018. 10.1186/s12929-018-0453-1. 29801500 10.1186/s12929-018-0453-1 PMC5968583

[28] Wu Y, Xie Q, Wu L, Li Z, Li X, Zhang L, Zhang B. Identification of activating transcription factor 6 (ATF6) as a novel prognostic biomarker and potential target in oral squamous cell carcinoma. Gene. 2024. 10.1016/j.gene.2024.148436. 38579904 10.1016/j.gene.2024.148436

[29] Yuan Y, Jiao P, Wang Z, Chen M, Du H, Xu L, Xu J, Dai Y, Wu F-G, Zhang Y, Wu H. Endoplasmic reticulum stress promotes the release of exosomal PD-L1 from head and neck cancer cells and facilitates M2 macrophage polarization. Cell Commun Signal. 2022. 10.1186/s12964-021-00810-2. 35090495 10.1186/s12964-021-00810-2 PMC8796490

[30] Zhang Q, Zhu XD, Sun QN, Wang D. The UPR signaling molecule ATF6 is a poor prognostic marker in gastric cancer. Asian J Surg. 2022;45:2836–7. 10.1016/j.asjsur.2022.06.057. 35835674 10.1016/j.asjsur.2022.06.057

[31] McMellen A, Yamamoto TM, Qamar L, Sanders BE, Nguyen LL, OrtizChavez D, Bapat J, Berning A, Post MD, Johnson J, Behbakht K, Nurmemmedov E, Chuong EB, Bitler BG. ATF6-mediated signaling contributes to PARP inhibitor resistance in ovarian cancer. Mol Cancer Res. 2023;21:3–13. 10.1158/1541-7786.Mcr-22-0102. 36149636 10.1158/1541-7786.MCR-22-0102 PMC9812934

[32] Li R, Zhou H, Li M, Mai Q, Fu Z, Jiang Y, Li C, Gao Y, Fan Y, Wu K, Costa CD, Sheng X, He Y, Li N. Gremlin-1 promotes colorectal cancer cell metastasis by activating ATF6 and inhibiting ATF4 pathways. Cells. 2022. 10.3390/cells11142136. 35883579 10.3390/cells11142136 PMC9324664

