# Supplementary material for: ATF6 activation promotes tumorigenesis and drug resistance in diffuse large B-cell lymphoma (DLBCL) by regulating the mTOR/S6K signaling pathway
Source: Discov Oncol. 2025 Apr 9;16:499. doi: 10.1007/s12672-025-02264-1 (PMC11982007; doi:10.1007/s12672-025-02264-1)
Supplement: Supplementary file 1 — Supplementary Material 1. [file 12672_2025_2264_MOESM1_ESM.docx]

Supplementary Material

Supplementary Methods

**Clinical specimens**

A total of 67 paraffin-embedded archival tissues were collected from DLBCL patients (37 females and 30 males; age range: 19 - 82 years old, median age: 62 years old) diagnosed in the Department of Hematology of the Fourth Hospital of Hebei Medical University between 2019 and 2022. Healthy donors were recruited to donate peripheral blood samples. Then the Ficoll-Hypaque density gradient centrifugation method was used to extract peripheral blood mononuclear cells (PBMCs). Informed consent was obtained from each human sample in strict compliance with the Declaration of Helsinki. All study protocols were reviewed and approved by the Medical Ethical Committee of The Fourth Hospital of Hebei Medical University.

**Immunohistochemistry (IHC) staining and results interpretation**

The paraffin in paraffin-embedded tissue samples that measured 4-μm-thick were first removed and samples were rehydrated. 1x Sodium citrate buffer was then used to retrieve antigens under high pressure followed by a 1.5h cooling period. 3% hydrogen peroxide was then used to block endogenous peroxidase reactions before the samples were incubated with 5% BSA to nullify non-specific. Tissue sections were further left to incubate overnight with primary antibodies, anti-ATF6 (1:300, Abcam), anti-pS6K (1:70, Abcam) at 4℃. The slides were then rinsed the next morning before being allowed to incubate for another 30 minutes with a secondary antibody at 37℃. The slides were then stained with diaminobenzidine (DAB), counterstained with hematoxylin and mounted. Two independent pathologists assessed the staining intensity and percentage of positive cells in all the sections in a double-blind manner. The staining intensity was categorized into four levels: 0 (negative), 1 (weak), 2 (moderate), and 3 (strong). The percentage of positive tumor cells was categorized into four levels: 1 (≤25%), 2 (26-50%), 3 (51-75%), and 4 (>75%). Immunoreactivity score (IRS) was calculated using the following equation: IRS = staining intensity × percentage of positive cells.0-3 indicates “Negative”; 4-12 indicates “Positive”.

**Flow Cytometry Analysis**

Apoptosis in the treated DLBCL cells was analyzed by flow cytometry. DLBCL cells were treated with 20 μM ceapinA7 and siATF6 for 24h. The cell pellets were collected by centrifugation, washed with PBS, and centrifuged again. The cell pellet was resuspended in buffer at a density of 1 × 10^6 cell/ml, stained with Annexin-V and PI. The samples were then incubated in the dark at ambient temperature for 15 min and analyzed using a Navios flow cytometer (Beckman Coulter, USA).

**ATF6 silencing**

ATF6 was knocked down by siRNA transfection in a human DLBCL cell line. Cells were cultured in 6-well plates at a density of 1 x 10^6 cells/well, with 2 ml per well. The next day, siRNA was transfected using CALNPTM RNAi in vitro (D-Nano Therapeutics, China) transfection reagent according to the manufacturer's instructions. A generic and nonsensical RNA sequence was used as the negative control siRNA. The effectiveness of the transfection was confirmed by western blot and qRT-PCR assays.

**Cytotoxicity Assay**

Cell viability was assessed using the Cell Counting Kit-8 (CCK-8, Beyotime, China) assay. Drug-treated DLBCL cell suspensions were inoculated in 96-well plates for 24-48 hours. Subsequently, 10 μl of CCK-8 was added to each well, and incubated for 2h at 37°C, and the absorbance at 450 nm was measured using a Multiskan GO plate reader (Thermo Scientific, USA).

**Western blotting**

After all experimental procedures, DLBCL cells were collected, washed, and lysed in lysis buffer (Servicebio, China) together with 1× final concentration of phosphatase inhibitor cocktail (PhosSTOP; MCE, USA). Equal protein extracts (30μg) were then separated using FuturePAGE™ Protein Prep Gel (ACE, China) and electro blotted onto polyvinylidene fluoride (PVDF) membranes (Millipore, USA). The membranes were blotted for 2 hours with 5% non-fat powdered milk at room temperature, and then subsequently blotted overnight at 4°C with the indicated antibodies. The following day, the PVDF membraes were TBST-rinsed and hybridized for 1 hour at room temperature with HRP-conjugated secondary antibodies (Proteintech, China). Chemiluminescent signals were evaluated with the electro-chemi-luminescence kit (Coolaber, USA) with the ChemiDoc XRS+ System (BIO-RAD, USA).
